# Characteristics of non-AIDS-defining malignancies in the HAART era: a clinico-epidemiological study

**DOI:** 10.1186/1758-2652-14-16

**Published:** 2011-03-28

**Authors:** Nicolas Dauby, Stéphane De Wit, Marc Delforge, Valentina Coca Necsoi, Nathan Clumeck

**Affiliations:** 1 Division of Infectious Diseases, CHU St-Pierre, Université Libre de Bruxelles, Brussels, Belgium

## Abstract

**Background:**

Non-AIDS-defining malignancies (NADM) are becoming a major cause of mortality in the era of highly active antiretroviral therapy. We wished to investigate the incidence, risks factors and outcome of NADM in an urban cohort.

**Methods:**

We carried out an observational cohort of HIV patients with 12,746 patient-years of follow up between January 2002 and March 2009. Socio-demographics and clinical characteristics of patients diagnosed with NADM were retrospectively compared with the rest of the cohort. Causes of death and risk factors associated with NADM were assessed using logistic regression. Survival analyses were performed with Kaplan-Meier estimates. Cancer incidences were compared with those of the general population of the Brussels-Capital Region using the standardized incidence ratio (SIR).

**Results:**

Forty-five NADM were diagnosed. At inclusion in the study, patients with NADM were older than patients without NADM (47 years vs. 38 years, p < 0.001), had a longer history of HIV infection (59 months vs. 39 months, p = 0.0174), a lower nadir CD4 count (110 cells/mm^3 ^vs. 224 cells/mm^3^, p < 0.0001) and a higher rate of previous AIDS events (33% vs. 20%, p = 0.0455) and of hepatitis C virus co-infection (22.2% vs. 10%, p = 0.0149). In multivariate analysis, age over 45 at baseline (OR 3.25; 95% CI 1.70-6.22) and a nadir CD4 count of less than 200 cells/mm^3 ^(OR 3.10; 95% CI 1.40-6.87) were associated with NADM. NADM were independently associated with higher mortality in the cohort (OR 14.79; 95% CI 6.95-31.49). Women with cancer, the majority of whom were of sub-Saharan African origin, had poorer survival compared with men. The SIR for both sexes were higher than expected for Hodgkin's lymphoma (17.78; 95% CI 6.49-38.71), liver cancers (8.73; 95% CI 2.35-22.34), anal cancers (22.67; 95% CI 8.28-49.34) and bladder cancers (3.79; 95% CI 1.02-9.70). The SIR for breast cancer was lower in women (SIR 0.29; 95% CI 0.06-0.85).

**Conclusions:**

Age over 45 and a nadir CD4 count of less than 200 cells/mm^3 ^were predictive of NADM in our cohort. Mortality was high, especially in sub-Saharan African women. Cancers with increased incidences were Hodgkin's lymphoma and anal, bladder and liver cancers in both sexes; women had a lower incidence of breast cancer.

## Background

HIV infection is associated with an increased incidence of certain types of cancer, i.e., Kaposi sarcoma, non-Hodgkin's lymphoma and cervical cancer, all linked with profound immunosuppression [1]. These cancers have been classified as AIDS-defining malignancies (ADM) by the Centers for Disease Control and Prevention since 1993.

The introduction of highly active antiretroviral therapy (HAART) led to a change in the causes of hospitalization and death of HIV-infected patients, with a significant decrease in AIDS-related causes, like ADM, but with a rise in cardiovascular diseases and non-AIDS-defining malignancies (NADM); these became major causes of mortality in the HAART era [2-4].

Various registry-linked epidemiological studies have stressed an increased risk of malignancies in HIV patients in the era of HAART in comparison with the general population [5-7]. Causative factors were first thought to be a higher proportion of risk factors in the HIV populations, such as tobacco smoking or intravenous drug use. However, more data are now pointing to the role of prolonged immunosuppression, independently of other risk factors.

A meta-analysis comparing the incidence of cancer in transplant patients and HIV-infected patients demonstrated that in terms of cancer risk factors, these two distinct populations have increased incidences of various neoplasms, such as Hodgkin's lymphoma, anal cancer, lung cancer and liver cancer [8]. Recent data have confirmed the impact of long-term immunosuppression, as assessed by nadir and current CD4 cell count on the occurrence of NADM [9].

We report our experience with NADM in a single-centre cohort of HIV-infected patients during the late HAART era. We sought to identify risks factors associated with the occurrence of NADM, to describe the characteristics of patients diagnosed with a NADM and to compare the incidence of these cancers in our cohort with the population of the Brussels-Capital Region. We also provide a descriptive analysis of cancers diagnosed during the study period, along with the treatments received, as well as their complications.

## Methods

### Study population

The Brussels St-Pierre HIV cohort is an urban cohort initiated in 1983 at the University Hospital St-Pierre, located in downtown Brussels, Belgium. Since 1983, it has accumulated data on more than 5000 patients. Socio-demographics characteristics (birth date, ethnicity), data about HIV infection (date of diagnosis, serial CD4 counts and viral loads, AIDS diagnosis), treatment (drugs, date of initiation or change, reason for change), co-infections with hepatitis B and C viruses, and occurrence of adverse events (cancer, opportunistic infection, hospitalization, death) are prospectively collected and encoded in a database.

Patients are recruited through doctor referral from the outpatient and inpatient HIV units. In 2009, there were 2302 patients under active follow up, made up of 59% males, 49% Caucasians and 47% sub-Saharan Africans. Transmission origin was mainly heterosexual (57%) and homo/bisexual (32%), while intravenous drug use accounted for only 4%.

For the present study, patients who were diagnosed with a NADM between 1 January 2002 and 31 March 2009 were retrospectively compared with the rest of the cohort (e.g., those who did not have a diagnosis of NADM). Data about demographics, mode of transmission and HIV characteristics were retrieved from the database and comparisons were made between the two groups. Data were retrieved from the database for patients who met the following criteria: age over 18, and at least two contacts in the outpatient clinic or one hospitalization and at least one contact in the outpatient clinic during the study period.

A retrospective review of the clinical charts of the patient with NADM was performed and the following data were retrieved: symptoms; method of diagnosis; treatment received (surgery, chemotherapy, radiotherapy, other); complications observed for each treatment; and occurrence of any opportunistic infection in chemotherapy recipients within 12 months of initiation of the treatment. The study was approved by the local ethical review committee of the St-Pierre Hospital.

### Statistical analysis

Categorical data were compared with Fisher or Chi-square tests. Continuous variables were compared with the non-parametric Mann-Whitney test. Risks factors associated with death and NADM diagnosis were assessed using logistic regression with calculation of odds ratio (OR). For NADM patients, Kaplan-Meier analyses were made since the time of cancer diagnosis.

Statistical analysis were performed with MedCalc for Windows, version 9.5.0.0 for the Kaplan-Meier analysis (MedCalc Software, Mariakerke, Belgium), GraphPad Prism version 5.01 for Windows for analysis of the continuous and categorical data (GraphPad Software, San Diego, California, USA), and Stata 11 (StatCorp LP, College Station, Texas, USA) for the logistic regression.

### Calculations of the standardized incidence ratio

The expected numbers of cancers were calculated by multiplying the person-years at risk by the appropriate age- and gender-specific incidence rates, which were obtained from the Belgian Cancer Registry (http://www.registreducancer.be/) for the year 2005 (middle of the study period). We used data from the Brussels-Capital Region where our centre is located. The region is the largest urban centre of Belgium, with a population of about 1.1 million, and has the highest proportion of foreigners in Belgium (almost 30%) [10]. People from the Democratic Republic of Congo, which represent the main population of sub-Saharan African origin in Belgium, live mainly in the Brussels-Capital Region [11]. Sub-Saharan Africans account for about 12% of the legally registered foreign population of the region [10].

The standardized incidence ratio (SIR) was calculated using the ratio of observed to expected numbers of cancer cases, and 95% confidence intervals (CIs) were calculated. Non-melanoma skin cancers were excluded because they were at risk of being under-reported and are not associated with high morbidity or mortality.

## Results

A total of 3126 patients who met the inclusion criteria were included, with 12,746 patient-years of follow up. Forty-five NADM were diagnosed during the study period (Table 1). Two cases of NADM were excluded from the analysis: one breast cancer because the diagnosis of HIV was made after the diagnosis of cancer; and one thyroid cancer because the diagnosis of cancer was made abroad and available data were insufficient. Of note, three patients diagnosed with NADM were previously diagnosed with an ADM: one with non-Hodgkin's lymphoma; and two with Kaposi sarcoma.

**Table 1:** Non-AIDS-defining malignancies distribution during the study period

Cancer type	n	%
Hodgkin's lymphoma	6	13.3
Anal	6	13.3
Lung	5	11.1
Hepatocellular carcinoma	4	8.9
Prostate	4	8.9
Bladder	4	8.9
Breast	3	6.7
Head & neck	3	6.7
Others	10	22.2
		
All	45	100

Others: vagina, stomach, testicular, oesophagus, acute leukemia, colon, kidney, skin melanoma, vulva and ovary cancer

### Patients characteristics

Characteristics of the NADM patients compared with the rest of the cohort are summarized in Table 2. Patients diagnosed with NADM were older and had a longer history of HIV infection. There were no differences in the proportions of males, Africans, men who have sex with men (MSM) and smokers. A two-fold higher rate of hepatitis C virus co-infection was noted in the NADM group. When considering CD4 cell count at inclusion in the study, no difference was noted, but nadir CD4 count was lower for NADM patients. Both groups had a similar proportion of subjects on HAART at inclusion, and the cumulative exposure to HAART was not statistically different between the two groups. An 18-fold increase of mortality was observed for the NADM group. In a multivariate analysis (Table 2) using logistic regression, the two factors independently associated with the risk of NADM during the study period were age over 45 at baseline and a nadir CD4 count of less than 200 cells/mm^3^.

**Table 2:** Characteristics of the patients diagnosed with a non-AIDS-defining malignancy compared with the cohort.

	Cohort		NADM		No NADM		Univariate			Multivariate		
	
	n	%	n	%	n	%	p value	OR	CI 95%	p value	OR	CI 95%
Patients	3126	100	45	1.4	3081	98.6						
Included after 1 January 2002	1300	41.6	10	22.2	1290	41.9						
Median follow up	49		60		49							
Age > 45 at baseline	703	22.49	24	53.33	679	22.04	**< 0.0001**	**4.04**	**2.24-7.31**	**< 0.0001**	**3.25**	**1.70-6.22**
Smoking	984	31.5	19	42.2	965	31.3	0.1611					
MSM	875	28.0	11	24.4	864	28.0	0.714					
Male	1791	57.3	30	66.7	1761	57.2	0.2004					
African origin	1582	50.6	19	42.2	1563	50.7	0.3255					
												
HCV	319	10.2	10	22.2	309	10.0	**0.0149**	**2.56**	**1.3 - 5.2**	0.057	2.10	0.98-4.53
HBV	192	6.1	4	8.5	188	6.1	0.6453					
												
HIV diagnosis >5 years	1154	36.9	21	46.67	1133	36.77	0.1722					
Nadir CD4 count (cells/mm^3^)	220		110		224							
Nadir CD4 count < 200/mm^3 ^before 1 January 2002	1410	45.10	35	77.78	1375	44.63	**< 0.0001**	**4.34**	**2.14-8.80**	**0.005**	**3.10**	**1.40-6.87**
CD4 count at baseline (cells/mm^3^)	402		375.5		405		0.2782					
VL at baseline	559		326		573		0.6767					
												
HAART use at baseline	78.0		71.4		78.1		0.7514					
Cumulative duration of HAART	39		50		38		0.1201					
Time since initiation of HAART	45		55		44		**0.0279**					
												
AIDS events before inclusion	635	20.31	15	33.3	620	20.12	**0.0455**	**1.98**	**1.1 - 3.7**	0.28	1.47	0.73-2.96
												
Death during study period	146	4.67	20	44.4	126	4.1	**< 0.0001**	**18.76**	**10.1 - 34.7**			

Multivariate analysis was performed using logistic regression. Durations are in months. VL: viral load. OR: odds ratio

When looking at the NADM group, at the time of cancer diagnosis, median CD4 count was 352 cells/mm^3 ^and 51.1% (n = 23) of the patients had undetectable viral loads (< 50 copies/ml). In patients with detectable viral loads, values ranged from 1330 to 482,000 copies/ml (median 87,100 copies/ml). Before cancer diagnosis, 53.3% of the patients had undetectable viral loads for at least six months. In the cancer group, differences were found between the two ethnic groups represented. Africans were mostly women, whereas Caucasians were mostly males (male:female ratio of 0.7 and 7.3, respectively; data not shown). Caucasian patients were more likely than African patients to be smokers (65.4 vs 25%, p < 0.01). Sex between men as a mode of transmission was frequent in the Caucasian population, but absent in the African population (42.3% vs 0%).

### Cancer, treatment characteristics and treatment complications

The majority of patients (65%) were symptomatic at diagnosis, while 13% were diagnosed after a screening procedure and 15% after an imaging diagnostic procedure for unrelated disease in asymptomatic patients. Of note, all prostate cancers were diagnosed after prostate-specific antigen testing (data not shown). The other cancers diagnosed after screening were hepatocellular carcinoma (alpha-foeto protein testing, n = 1) and breast cancer (mammography, n = 1). Histopathology was available in 42 cases (91%), and non-surgical biopsy was the most used tool (n = 31; 67%).

Treatments received were as follows: chemotherapy (54.3%, n = 25), surgery (41.3%, n = 19), radiotherapy (43.5%, n = 20) and hormonotherapy (8.7%, n = 4). Non-AIDS-related infections were the most common complications in chemotherapy recipients (32%, n = 8), with febrile neutropenia (n = 5) the most frequent. Opportunistic infections within 12 months of initiation of chemotherapy were observed in five patients (20%): lung tuberculosis (n = 1), lung *Mycobacterium xenopi *infection (n = 1), lung aspergillosis (n = 1), shingles (n = 1) and oesophageal candidiasis along with shingles (n = 1). Haematological complications (neutropenia, severe anemia, pancytopenia) were the second most common complications and occurred in 28% of the patients (n = 7).

Renal failure was the most common metabolic complication (n = 3), followed by toxic cardiopathy (n = 1) and chemotherapy-related pneumonitis (n = 1). Post-operative complications occurred in 31.6% (n = 6) of patients who underwent surgical procedures: small-bowel occlusion, bladder perforation, bacterial pneumonia, *Enterobacter cloacae *peritonitis along with two deaths (one following resection of a brain metastasis of breast cancer and the other after hepatectomia in a patient with liver cancer). Radiotherapy complications occurred in 25% of the cases (n = 5) and were localized dermatitis in all cases, except for one case of herpetic infection.

### Causes of mortality in the cohort and survival after cancer diagnosis

**Figure 1:**
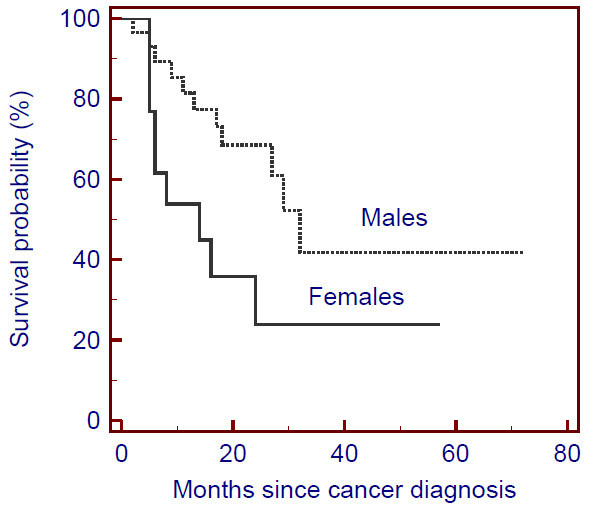
**Survival after cancer diagnosis**. Survival after cancer diagnosis is lower for women compared to men (14 vs 32 months; p = 0,037, Logrank test).

We used logistic regression to assess the causes of mortality during the study period (Table 3). Multivariate analysis revealed that NADM was independently associated with mortality, with an odds ratio (OR) of 14.79. After diagnosis of cancer, mean survival was 27 months (data not shown). Females had a lower survival in comparison with males (14 vs. 32 months, p = 0.037; HR = 3.004; 95% CI 1.069-8.442) (Figure 1).

**Table 3:** Analysis of the causes of death during the study period using logistic regression.

	Univariate				Multivariate			
Factor	OR	95% CI		p value	OR	95% CI		p value
**Nadir CD4 count <200 cells/mm^3^**	**6.46**	4.18	10.00	< 0.001	**4.41**	2.57	7.56	< 0.001
**HCV positive**	**3.17**	2.12	4.74	< 0.001	**2.37**	1.51	3.71	< 0.001
**HbS Ag positive**	1.70	0.98	2.97	0.061				
**NADM diagnosis**	**18.46**	9.99	34.1	< 0.001	**14.79**	6.95	31.49	< 0.001
**Previous AIDS diagnosis**	**5.29**	3.78	7.41	< 0.001	**3.21**	2.16	4.77	< 0.001
**Age over 45**	**2.63**	1.87	3.68	< 0.001	**2.10**	1.41	3.11	< 0.001
**MSM**	**0.51**	0.33	0.79	0.002	0.66	0.40	1.09	0.108
**Time since HIV diagnosis >5 years**	**2.71**	1.93	3.8	< 0.001	**1.63**	1.09	2.42	0.016

MSM: men who have sex with men. NADM: non-AIDS-defining malignancy. OR: odds ratio.

### Standardized incidence ratio

Higher than expected incidence rates were found for three cancers in males: Hodgkin's lymphoma (SIR 12.81; 95% CI 3.45-32.81), liver cancer (SIR 8.64; 95% CI 1.74-25.23) and anal cancer (SIR 41.14; 95% CI 13.26-96.00). For women, higher incidence rates were found for Hodgkin's lymphoma (SIR 65.37; 95% CI 13.14-191.00) and bladder cancer (SIR 12.11; 95% CI 1.36-43.72), while a lower incidence rate for breast cancer was observed (SIR 0.29; 95% CI 0.06-0.85). When considering both sexes, higher incidence rates were observed for Hodgkin's lymphoma (SIR 17.78; 95% CI 6.49-38.71), liver (SIR 8.73; 95% CI 2.35-22.34), bladder (SIR 3.79; 95% CI 1.02-9.70) and anal cancers (SIR 22.67; 95% CI 8.28-49.34). No difference in incidence was found in males for lung, prostate, head and neck, bladder and all cancers (excluding non-melanoma skin cancer), while in females no difference was found for lung, liver, anal and all cancers (excluding non-melanoma skin cancer).

## Discussion

NADM are becoming a significant concern in the management of chronically HIV-infected patients on HAART. Aging *per se *might be an explanation for the increased incidence of NADM, but epidemiological studies have shown that compared with the general population and after standardization for age, HIV-infected patients are at increased risk of certain types of cancer [6,12-14]. This is the case for cancer linked to chronic viral infections, such as anal cancer (Human papilloma virus), Hodgkin's lymphoma (Epstein-Barr virus) and liver cancer (HCV and HBV) [8], and also for epithelial cancers, such as lung cancer independently of a smoking history [15].

Despite a low number of cancer cases, we have been able to detect in our cohort a significantly higher incidence for three types of cancer in comparison with the general population of the Brussels-Capital Region after standardization for age: Hodgkin's lymphoma, anal cancer and liver cancer. The link between immunosuppression and these cancers is well known: they are indeed found in excess in both transplant patients and HIV patients [8].

We did not find an excess rate for lung cancer, one of the most frequent NADM in HIV patients [6]. An explanation might be the high incidence of lung cancer in the Belgian population in comparison with other European countries, especially in males [16], or the low proportion of intravenous drug use in our cohort, a known risk factor for lung cancer in HIV patients [17].

An intriguing finding is the lower incidence of breast cancer in HIV women. A large study involving 85,268 HIV-infected women in the USA has previously reported a lower SIR for breast cancer, independently of the CD4 count [18]. Similar results have been reported in studies in Europe and sub-Saharan African, both before and during the HAART era [19-21]. Data from the US have shown that HIV-infected women have more probability of undergoing a screening mammography, ruling out an underestimation of this cancer [22]. In our cohort, such an explanation could not be discarded as only one breast cancer out of three was diagnosed after a screening mammography. Interestingly, a recently published study has shown that women infected with CXCR4-tropic virus had lower breast cancer incidence compared with women with CCCR5-tropic virus [23]. The chemokine receptor CXCR4 is highly expressed by breast cancer cells and is involved in metastasis formation [24]. These epidemiological data corroborate *in vitro *experiments that have demonstrated that CXCR4-tropic virus interact with breast cancer cells and induce their apoptosis [25]. Unfortunately, data about virus tropism are not available for our patients.

We also noted an increased incidence of bladder cancer in women and in the combined sample of both women and men. The incidence of bladder cancer is increased in transplant patients but not in HIV patients [8]. Data regarding this cancer in HIV patients are scarce: a recent review of the published cases suggest a younger age at diagnosis and a predominance of males [26].

Interpretation of the SIR results is limited by the small numbers of cases and the use of the cancer incidence data from the Brussels-Capital Region, while patients of African origin are over-represented in our cohort in comparison with the general population.

Multivariate analysis allowed us to identify two predictors of NADM in our cohort, namely age over 45 and a nadir CD4 count of less than 200 cells/mm^3^. Aging is a known risk factor for cancer and has already been shown to be independently associated with NADM in other studies [27,28]. Immunodeficiency assessed by both nadir and current CD4 cell count [9,12,29,30] has been shown to predict the occurrence of NADM in various studies. A recent prospective French study involving 52,278 patients followed up between 1998 and 2006 has shown that at a CD4 count below 500 cells/mm^3^, the cancer risk (Hodgkin's lymphoma, liver and lung cancer) progressively increased: the highest risk was when CD4 count was below 50 cells/mm^3 ^[9]. The concept of "immunological surveillance" could be an explanation: the decrease of CD4+ T cells and other immune dysfunctions induced by HIV infection lead to a decrease of both the control of oncogenic viruses like EBV [31] and the destruction of malignant precursors [32]. Direct oncogenic effects of the virus itself could also be implied. Indeed, *in vitro *experiments suggest that the *tat *HIV protein could interfere with DNA repair mechanisms and apoptosis increasing the risk of malignant transformation in the host cells [33,34]. However, clinical studies are discordant about the risk of NADM and their relation to HIV viral load. In one study, uncontrolled viral replication (RNA >4 log10 copies/ml) was associated with the occurrence of non-AIDS severe clinical events, including NADM (9.5% of non-AIDS clinical events) [35]. In another study, an uncontrolled HIV-RNA level was associated with the occurrence of an ADM but not of a NADM [30]. We were unable to find such association in our study in multivariate analysis.

NADM are now a leading cause of death in HIV patients on HAART. In 2005 in France, 34% of deaths in HIV-positive patients were cancer related, 61% being non-AIDS related [36]. In our study, the diagnosis of NADM during the study raised the mortality by up to 15-fold. Survival after cancer diagnosis was poor and women had a significantly lower survival rate than men. Women with NADM were mostly of African origin. Various arguments suggest a heightened vulnerability of this particular group. First, this group is known to experience socio-economic precariousness: 41% of HIV-infected women of sub-Saharan Africa origin who died in 2005 in France were living in socio-economic precariousness compared with 23% of women born in France [37]. In our centre, 86% of African women were living on less than 1000 euros per month [38]. In the latter study, the majority of them were widowed and had children, 15% of whom were also HIV infected. Psychological distress was also frequent, along with poor treatment adherence.

A more advanced disease at diagnosis, a lower performance status and a higher tumour grade account for the lower survival observed in HIV-positive patients in comparison with HIV-negative patients [1]. HIV-infected patients may also be at increased risk of complications during chemotherapy: the combination of HIV-induced immunodeficiency with the haematological and metabolic toxicities of anti-cancer agents, potentially enhanced by HAART [1,39], makes HIV patients at higher risk of infectious complications and poorer survival. Infectious complications in patients who received chemotherapy were found in our study in up to 30% of patients, and opportunistic infections were observed in 20% of patients who received chemotherapy within the 12 months following the first date of chemotherapy.

## Conclusions

In our study, NADM with increased incidences were anal cancer, bladder cancer, Hodgkin's lymphoma and liver cancer. Immunosuppression, as assessed by a nadir CD4 cell count of less than 200 cells/mm^3^, was independently associated with the occurrence of NADM, along with an age of over 45 years. Mortality was high, especially in women, and infectious complications were the most frequent. Women from sub-Saharan Africa seem to be particularly vulnerable, and a multidisciplinary approach is required in the management of these patients. Efforts to reduce cancer incidences should be focused on improving CD4 counts with adequate therapy, prevention of immunosuppression with earlier treatment initiation, lowering of cancer risk factors, and appropriate screening programmes for cancers where this strategy has been shown to be effective [40].

## Competing interests

The authors declare that they have no competing interests.

## Authors' contributions

ND participated in the study design, reviewed the clinical charts, performed data analysis and statistical analysis, and writing of the paper. SDW participated in the study design, data analysis, and writing of the paper. MD extracted the data, and performed part of the statistical analysis. VCN encoded the data, and was in charge of the cohort. NC participated in study design, and reviewed the draft. All authors have read and approved the final manuscript.
